# Effectiveness of Internet-Based Interventions for the Prevention of Mental Disorders: A Systematic Review and Meta-Analysis

**DOI:** 10.2196/mental.6061

**Published:** 2016-08-17

**Authors:** Lasse Sander, Leonie Rausch, Harald Baumeister

**Affiliations:** ^1^ Institute of Psychology Depatment of Rehabilitationpsychology and Psychotherapy University of Freiburg Freiburg Germany; ^2^ Medical Faculty Department of Medical Psychology and Medical Sociology University of Freiburg Freiburg Germany; ^3^ Institute of Psychology and Education Department of Clinical Psychology and Psychotherapy University of Ulm Ulm Germany

**Keywords:** prevention, systematic review, meta-analysis, mental disorders, Internet and mobile-based

## Abstract

**Background:**

Mental disorders are highly prevalent and associated with considerable disease burden and personal and societal costs. However, they can be effectively reduced through prevention measures. The Internet as a medium appears to be an opportunity for scaling up preventive interventions to a population level.

**Objective:**

The aim of this study was to systematically summarize the current state of research on Internet-based interventions for the prevention of mental disorders to give a comprehensive overview of this fast-growing field.

**Methods:**

A systematic database search was conducted (CENTRAL, Medline, PsycINFO). Studies were selected according to defined eligibility criteria (adult population, Internet-based mental health intervention, including a control group, reporting onset or severity data, randomized controlled trial). Primary outcome was onset of mental disorder. Secondary outcome was symptom severity. Study quality was assessed using the Cochrane Risk of Bias Tool. Meta-analytical pooling of results took place if feasible.

**Results:**

After removing duplicates, 1169 studies were screened of which 17 were eligible for inclusion. Most studies examined prevention of eating disorders or depression or anxiety. Two studies on posttraumatic stress disorder and 1 on panic disorder were also included. Overall study quality was moderate. Only 5 studies reported incidence data assessed by means of standardized clinical interviews (eg, SCID). Three of them found significant differences in onset with a number needed to treat of 9.3-41.3. Eleven studies found significant improvements in symptom severity with small-to-medium effect sizes (d=0.11- d=0.76) in favor of the intervention groups. The meta-analysis conducted for depression severity revealed a posttreatment pooled effect size of standardized mean difference (SMD) =−0.35 (95% CI, −0.57 to −0.12) for short-term follow-up, SMD = −0.22 (95% CI, −0.37 to −0.07) for medium-term follow-up, and SMD = −0.14 (95% CI, -0.36 to 0.07) for long-term follow-up in favor of the Internet-based psychological interventions when compared with waitlist or care as usual.

**Conclusions:**

Internet-based interventions are a promising approach to prevention of mental disorders, enhancing existing methods. Study results are still limited due to inadequate diagnostic procedures. To be able to appropriately comment on effectiveness, future studies need to report incidence data assessed by means of standardized interviews. Public health policy should promote research to reduce health care costs over the long term, and health care providers should implement existing, demonstrably effective interventions into routine care.

## Introduction

Mental disorders remain highly prevalent worldwide with lifetime prevalence rates varying between 12.0% in Nigeria and 47.4% in the United States [1]. In 2010, the largest contributor to years lived with disability were mental and behavioral disorders [2]. In addition to the high disease burden and premature mortality, mental disorders also represent a financial burden for both, people affected and society [3-5].

Because care and treatment options and results remain limited [5], the focus should be on the reduction of the incidence by prevention measures.

There are 3 different types of prevention. Universal prevention is focused on the general population (including those without special risk factors). The focus of selective prevention is on subgroups at risk of developing a (mental) disorder (increased risk compared with the average population), whereas indicated prevention targets subgroups who show subthreshold symptoms (ie, not fulfilling full diagnosis criteria) [6].

Regardless of the type of prevention, prevention measures should lead to a substantial reduction in the incidence of the target mental disorder. Consequently, for assessing the effectiveness of preventive interventions, an initial disorder-free target population is needed. In addition, current incidence data collected by means of standardized interviews (eg, Structured Clinical Interview for DSM Disorders [SCID], MINI) are required [7,8]. Together, this allows for calculating the incidence rate ratios (IRRs) and number needed to treat (NNT). The NNT indicates how many people would have to receive an intervention to prevent one new case of the target mental disorder (“effort”), whereas the IRR is an indicator of the “impact” of a preventive intervention [9].

Recent reviews and meta-analyses indicated that prevention of mental disorders is feasible and can lead to a substantial “impact,” that is, reduction of incidence rates of mental disorders [9-13].

The Internet as medium for delivery has been identified as an appropriate way to scale up preventive interventions [14,15], needs less effort in provision, and has several additional advantages over traditional (ie, face-to-face) prevention settings. Internet- and mobile-based interventions (IMIs) are flexible (participants can integrate them easily in their daily lives and work at their own pace) and anonymity might be appealing for those fearing stigmatization [16]. Furthermore, in the setting of limited health care resources, IMIs have been found to be cost-effective [17-19]. A large number of people can be reached as a result of decreased personnel and infrastructure costs, especially those in remote areas without easy access to health care services [20]. Considering the worldwide rapid growth of Internet usage during the last decade [21], health care services and particularly mental health professionals could benefit from IMIs as an alternative or supplement to existing and traditional interventions [22].

There are few reviews and meta-analyses to date summarizing empirical findings on IMIs for the prevention of mental disorders. The literature yields reviews on prevention of eating disorders (EDs) [23,24], and substance-related and addictive disorders [25-27] in adult populations. Regarding ED, the review by Schlegl et al [24] integrated a wide range of intervention and prevention trials. Concerning prevention, they presented a mixture of relapse prevention (2 studies), treatment of subthreshold ED (2 studies), and primary prevention trials. Unfortunately, the wide range of studies and outcome parameters did not allow for meta-analytical pooling. Beintner et al [23] focused on a single ED prevention program for students called StudentBodies.

With regard to content, substance-related and addictive disorders prevention programs are often focused on health behaviors and health promotion, rather than on psychotherapeutic variables [28,29]. In summary, none of the previously mentioned reviews summarizes and evaluates the existing literature of Internet-based interventions for the prevention of mental disorders in general and with a clear reference to the previously mentioned statistical criteria (initial disorder-free target population, reporting of incidence data by means of standardized interviews). Consequently, health care providers and public health policy makers are unable to gain an overview of the effectiveness of IMIs for the prevention of mental disorders. This systematic review and meta-analysis fills this gap in research as understudied disorder groups, intervention types, and populations are detected. It aims to (1) describe existing studies on Internet-based preventive interventions, (2) assess the quality of included studies, (3) evaluate the intervention effectiveness, and (4) highlight understudied subfields of research (eg, certain disorder groups or intervention content).

## Methods

### Registration and Study Protocol

This systematic review has been registered in the PROSPERO register (registration number CRD42015026781). It was conducted according to the PRISMA guidelines [30]. A study protocol that describes trial details has been submitted on December 17, 2015 [31].

### Eligibility Criteria

#### Population

Studies were eligible for inclusion if they (1) focus on an adult target population, who (2) were without a diagnosis of the target mental disorder at baseline (primary prevention intervention). (3) Mental disorders had to be assessed by means of standardized interviews (eg, SCID [32]), validated self-reports (eg, Beck Depression Inventory-II [33]) or clinician-rated scales (eg, HAM-D [34] with normed cutoff points or diagnosed by health care professionals). Studies on the prevention of substance-related and addictive disorders have been excluded, as this represents a frequently-studied and already reviewed specific subgroup of prevention research [26,27].

#### Intervention

(4) Interventions needed to be based on psychological interventions. The definition of “psychological intervention” was taken from Kampling et al [35] and refers to cognitive behavioral therapy (CBT), psychodynamic psychotherapy, behavior therapy or behavior modification, systemic therapy, third wave cognitive behavioral therapies, humanistic therapies, integrative therapies, and other psychological-oriented interventions. (5) Interventions must be provided in an online setting, defined as online, Internet, Web, or mobile based. Interventions may vary concerning the amount of external guidance provided to participants. Self-help interventions will also be included. We excluded studies on the relapse prevention of mental disorder, as these treatment maintenance interventions differ substantially from preventive interventions focused on the first or recurrent onset of mental disorders [35].

#### Comparison

(6) Studies had to include a control group. This could be either (enhanced) usual care, wait-list control group, another intervention, or no treatment.

#### Outcomes

(7) Studies examining onset of disorder were included, defined as percentage of persons who developed the mental disorder under study from pre- to follow-up-assessment. In addition to data from standardized clinical interviews (eg, SCID-IV [7]), we included studies reporting only symptom severity scores, when validated rating scales with normed cutoff points (referencing onset of disorder or diagnosis) have been used. To be able to comment meaningfully on any postintervention reduction of incidence, studies had to (8) include a follow-up assessment at 3 months or longer after randomization.

#### Study Type

(9) Only randomized controlled trials (RCTs) that are available in full text will be eligible for this review. For an overview of the eligibility criteria, see Table 1.

**Table 1:** Eligibility criteria.

No.	Item	Inclusion	Exclusion
1	Population	Adults (≥ 18 years)	Children and adolescents (< 18 years)
2	Prevention	Universal, selective, or indicated prevention	Parts of the population already affected at baseline
3	Assessment	Instrument with standardized cut-offs for clinical significance or symptom severity (> moderate symptomatology)	Descriptive symptom-oriented instruments without standardized cut-offs
4	Prevented disorder	Mental disorder other than substance-related/addictive disorder	Other types of disorders; substance-related/addictive disorders
5	Intervention	Web-based, psychological, preventive	Not Web-based, no psychological principles, treatment rather than prevention
6	Control group	Waiting list, other treatment, placebo, care as usual	No control group
7	Outcomes	Onset, Number Needed to Treat, Incidence Rate Ratio, severity	Other outcomes (no statements possible about preventive effect)
8	Follow-up	At least 3 month follow-up assessment	No or < 3-month follow-up assessment
9	Study design	Randomized controlled trial	No randomized controlled trial (eg, cross-sectional studies, case studies, or case reports)

### Search Strategy

A systematic database search was conducted. Databases included are The Cochrane Central Register of Controlled trials (CENTRAL), PsycINFO, and MEDLINE (search date August 17, 2015). A sensitive search strategy was developed and applied for each database [31]. The search was complemented by a review of reference lists from identified publications and a hand-search of the World Health Organization International Clinical Trials Registry Platform (ICTRP) to include ongoing trials.

When indicated, study authors have been contacted to obtain further information to clarify study characteristics. When study protocols were identified without subsequent publication of results, authors have been contacted to obtain missing or unpublished data and determine eligibility for inclusion in this review.

### Study Selection

**Figure 1:**
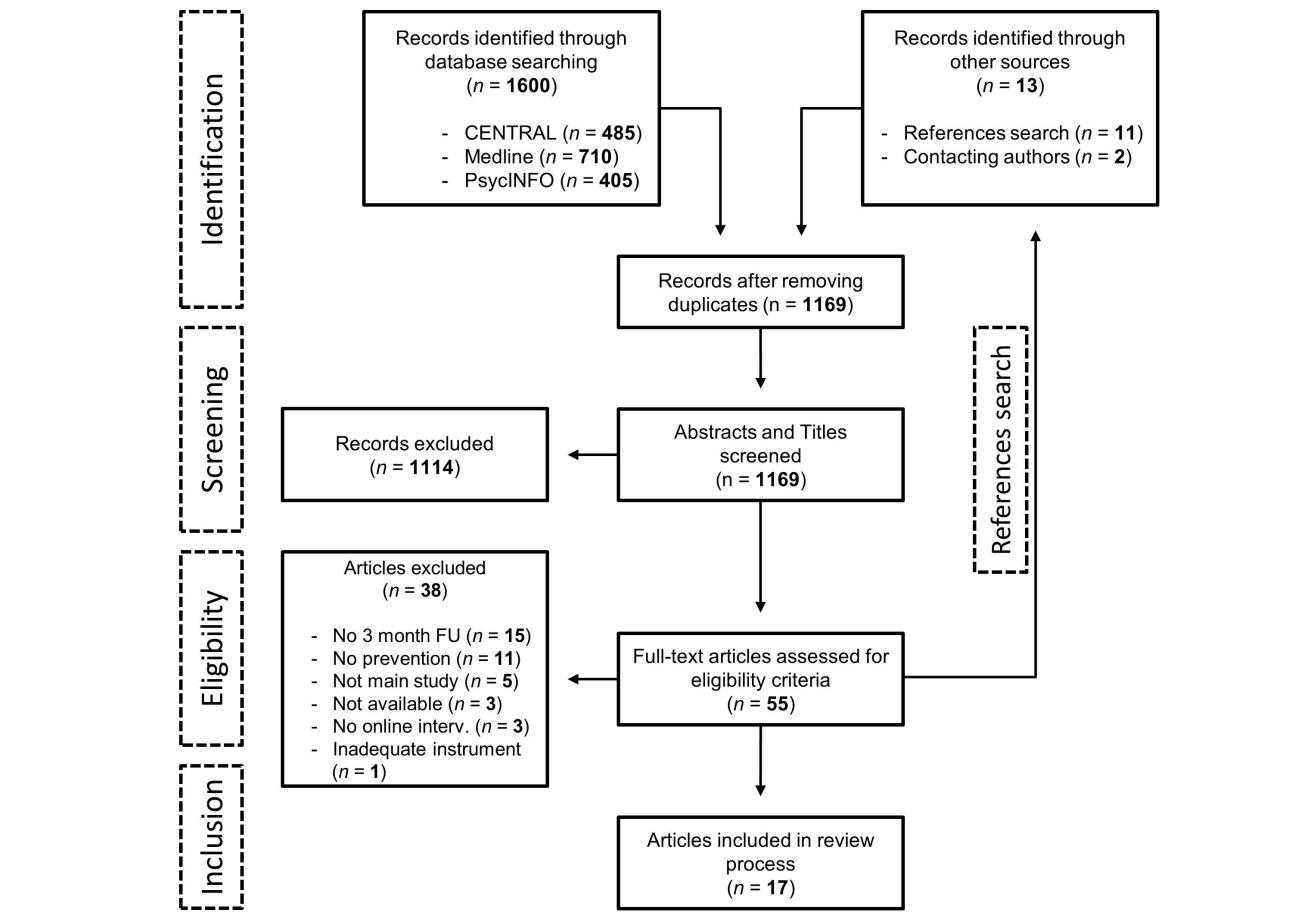
PRISMA (Preferred Reporting Items for Systematic Reviews and Meta-Analyses) flow chart of included studies.

The selection of papers was conducted by 2 independent reviewers (LS, LR). In the first step, authors screened all titles and abstracts yielded by the database search. In the second step, the full texts of the selected articles were retrieved and screened in terms of the aforementioned eligibility criteria. Reference lists of all articles included in the study were screened in the same way. Disagreement at both screening levels was resolved by discussion. Concurrent validity of the 2 reviewers was examined. Figure 1 shows a PRISMA flow chart to illustrate the study selection process and reasons for exclusion [30].

### Data Extraction

The following data items were extracted for each study: (1) study identification items (first author, year of publication), (2) study design characteristics (sample size, control group, type of assessments, length of follow-up assessments), (3) intervention characteristics (name, type, duration, level of human support or guidance), (4) prevention characteristics (type, prevented disorder), (5) dropout rate, (6) target population (eg, risk group), and (7) clinical outcomes (onset and/or severity of disorder including means, variances, as well as effect sizes). In case of deficient or missing outcome data, authors were contacted and data were requested. To ensure accuracy, a second reviewer rechecked the extracted data.

### Assessment of Methodological Quality

To evaluate the quality of evidence, the risk of bias was assessed for each study according to the Cochrane Collaboration’s tool for assessing risk of bias in RCTs [36]. The assessment was rechecked by a second reviewer. As recommended, each study was reviewed for procedures in the following domains: (1) random sequence generation, (2) allocation concealment, (3) blinding (3a – of participants, 3b – of personnel, 3c – of outcome assessors), (4) incomplete outcome data (4a – dropout rate (≤ 20% for short-term follow-ups, ≤ 30% for long-term follow-ups), 4b – intention-to-treat analysis), (5) selective outcome reporting, and (6) other threats to validity (6a – similar groups at baseline, 6b – no or similar cointerventions between intervention and control groups, 6c – compliance, 6d – identical timing for outcome assessment). Studies were rated as showing a “low” or “high” risk of bias according to the aforementioned criteria. For studies with at least 6 fulfilled criteria and no serious flaws, the risk of bias was evaluated as being low according to Furlan et al [36]. Less than 6 fulfilled criteria or serious flaws yielded a rating of “high” risk of bias. Of note, in the implementation of psychological interventions, blinding of health care providers (if a guided intervention was provided) or patients concerning the treatment is not possible. This results in a “high” risk of bias rating on this criterion. However, outcome assessors can remain unaware of the treatment allocation of patients.

### Data Analysis

If onset data were available, IRR and NNT were calculated. When there were at least 5 studies with available severity data within one disorder (as primary and secondary outcome), a meta-analytically pooled effect size was calculated, and effect sizes were illustrated in forest plots. Meta-analyses were conducted using Review Manager 5.3 (Cochrane Collaboration, 2014). Standardized mean differences (SMDs) with 95% CIs were computed for all continuous outcomes. Random-effects meta-analyses were performed to compute overall estimates of treatment outcome. The *I*^2^statistic was used to examine study heterogeneity [37]. Consistent with Sterne et al [38], a funnel plot examining publication bias was not examined due to the limited number of included studies. Follow-up periods were subgrouped into short-term (post assessment), medium-term (≤ 6 month), and long-term (> 6 month) follow-ups. Subgroup comparisons were not feasible due to the low number of studies included.

## Results

### Overview

The systematic database search yielded 1600 hits. After removing duplicates, screening titles, abstracts, and full text papers for inclusion, conducting a reference search, searching trial registers for eligible studies and contacting authors, a total of 17 studies met eligibility criteria and were included in the review. The selected studies targeted the prevention of EDs, depression, anxiety, post-traumatic stress disorder, generalized anxiety disorder (GAD), or a combination of these mental disorders [39-56].

### Quality Assessment

Five of 17 studies were classified as having a high risk of bias and the remaining 12 studies were classified as having a low risk of bias (Table 2). Sequence generation (1) was mostly met; only 3 studies were categorized as unclear. Allocation concealment (2) was met in almost half of the included studies, otherwise categorized as unclear as it was not sufficiently specified. Blinding of participants (3a) was met in 2 studies with active control groups [42,50]. Blinding of personnel (3b) was set on “no” without exception. As blinding of personnel is not possible for most psychological interventions, it must be considered a possible source of bias. However, outcome assessors remained unaware of the treatment allocation (3c) in 4 studies [40,42,43,51].

Regarding dropout rate (4a), only 7 studies met the predetermined criterion (≤20% for short-term follow-ups, ≤ 30% for long-term follow-ups). Ten of the included studies reported the use of an intention-to-treat analysis (4b); the remaining studies were categorized as unclear. Two studies reported results incompletely [39,48]. (5) Particularly for Jacobi et al [39] this can be regarded as serious flaw, since results of a clinical interview (SCID) were not reported. About half of the studies reported similar groups at baseline (6a) in demographics and outcome measures. Some studies reported differences in one demographic variable, which was subsequently used as a covariate [54]; alternatively, if the manuscript did not address this variable, the study was categorized as unclear. Two serious flaws were catalogued on this category because of disregarded baseline differences in outcome measures [43,53]. There were no cointerventions (6b) in any study. Compliance with interventions (6c) was rated acceptable in two thirds of the included studies. One study [50] received a serious flaw because of a very low compliance and significant between groups difference (combined with a relatively high dropout rate). (6d) Outcome assessment was timed similarly for all groups except for 2 studies [43,46] that used a cohort design.

To evaluate the risk of certain biases (selection, performance, detection, and attrition bias), the criteria can be grouped into randomization, blinding, outcome, and withdrawal criteria. Selection bias could only be ruled out in 3 studies [41,47,52] that fulfilled all 3 randomization criteria (1) sequence generation, (2) allocation concealment, (6a) similar groups.

Performance bias could be present in every study. As mentioned previously, blinding was only insufficiently possible for all studies. Hence, criteria concerning expectation and performance effects (3a – blinding of participants, 3b – blinding of personnel, 6b – no cointerventions, 6c – compliance) were never completely fulfilled.

Concerning detection bias, 3 studies [40,42,51] fulfilled both criteria of outcome assessment (3c – blinding of outcome assessors, 6d – identical timing for outcome assessments).

Three studies [45,47,54] fulfilled both criteria concerning withdrawals from studies (4a – dropout, 4b – ITT); the remaining studies could be affected by attrition bias.

### Intervention Characteristics

Many studies administered measures of a variety of mental disorders. Hereinafter included studies are arranged by their main focus. For an overview of all included studies, see Table 3.

**Table 2:** Risk of bias assessment.

Study	Sequence generation^a^	Allocation concealment^c^	Blinding			Incomplete outcome data		Selective outcome reporting^i^	Other threats to validity				Risk of Bias^n^
			Participants^d^	Personnel^e^	Outcome assessors^f^	Dropout^g^	ITT^h^		Similar groups^j^	Cointerventions^k^	Compliance^l^	Timing^m^		
Bantum et al [46]													
	Yes^b^	Unclear	No	No	No	Yes	Unclear	Yes	No	Yes	Yes	No	High	
Beatty et al [47]													
	Yes	Yes	Unclear	No	No	Yes	Yes	Yes	Yes	Yes	No	Yes	Low	
Christensen et al [48]													
	Yes	Yes	Unclear	No	No	No	Yes	No	No	Yes	Yes	Yes	Low	
Christensen et al [55]													
	Yes	Yes	No	No	Yes	No	Yes	Yes	Yes	Yes	Yes	Yes	Low	
Imamura et al [49]													
	Yes	Yes	No	No	No	No	Yes	Yes	Unclear	Yes	No	Yes	Low	
Jacobi et al [39]													
	Yes	Unclear	No	No	No	Yes	Unclear	No	Unclear	Yes	Yes	Yes	High^o^	
Jacobi et al [40]													
	Yes	Unclear	No	No	Yes	No	Unclear	Yes	Unclear	Yes	Yes	Yes	Low	
Mitchell et al [50]													
	Yes	Yes	Yes	No	No	No	Yes	Yes	Unclear	Yes	No	Yes	High^p^	
Mouthaan et al [51]													
	Yes	Yes	No	No	Yes	No	Yes	Yes	Unclear	Yes	Yes	Yes	Low	
Musiat et al [41]													
	Yes	Yes	No	No	No	No	Unclear	Yes	Yes	Yes	No	Yes	Low	
Powell et al [52]													
	Yes	Yes	No	No	No	No	Yes	Yes	Yes	Yes	No	Yes	Low	
Proudfoot et al [53]													
	Yes	Yes	No	No	No	No	Yes	Yes	No	Yes	No	Yes	High^q^	
Stice et al [42]													
	Yes	Unclear	Yes	No	Yes	Yes	Unclear	Yes	Yes	Yes	Yes	Yes	Low	
Taylor et al [43]													
	Yes	Unclear	No	No	Yes	Yes	Unclear	Yes	No	Yes	Yes	No	High^r^	
Thompson et al [54]													
	Unclear	Unclear	No	No	No	Yes	Yes	Yes	Yes	Yes	Yes	Yes	Low	
Winzelberg et al [44]													
	Unclear	Unclear	No	No	No	No	Yes	Yes	Yes	Yes	Yes	Yes	Low	
Zabinski et al [45]													
	Unclear	Unclear	No	No	No	Yes	Yes	Yes	Yes	Yes	Yes	Yes	Low	

^a^Random unpredictable assignment sequence.

^b^Yes = Criterion has been met (low risk of bias); No = Criterion has not been met (high risk of bias)

^c^Assignment generated by an independent person who is not responsible for determining the eligibility of participants.

^d^Intervention and control group are indistinguishable for the participants.

^e^Intervention and control group are indistinguishable for the care providers.

^f^Intervention and control group are indistinguishable for the outcome assessors (for patient reported outcomes, it is adequate if patients are blinded).

^g^Dropout must be described and reasons must be given, for short term follow-ups (eg, 3 months) 20%, for long term follow-ups (eg, ≥ 6 months) 30% should not be exceeded.

^h^ITT: intention-to-treat; all randomized patients are reported and analyzed in the group they were allocated to by randomization.

^i^Results of all pre-specified outcomes have to be adequately and completely reported.

^j^Groups should not differ significantly at baseline regarding demographics and outcomes.

^k^There are no cointerventions or they are similar between intervention and control groups.

^l^Acceptable compliance with the intervention (eg, intensity, duration, number, frequency of sessions).

^m^Identical timing of outcome assessments for intervention and control groups.

^n^≥ 6 x “Yes” and no serious flaws indicates an overall low risk of bias; < 6 x “Yes” or serious flaws indicates an overall high risk of bias.

^o^Serious flaw: Results of diagnostic interviews not reported.

^p^Serious flaw: Very high dropout and very low compliance rate.

^q^Serious flaw: Baseline differences between groups, very low compliance.

^r^Serious flaw: Baseline differences between groups in several scales.

**Table 3:** Characteristics of included studies

Study	Prevention type	Prevented disorder	Targeted population	Program name	Intervention type (duration)	Conditions	Sample size (n)	Instrument	Follow-up months	Drop-out^a^	ITT^b^
Bantum et al [46]											
	Selective	Depression	Adult cancer survivors	Surviving and Thriving with Cancer (STC)	Assisted health behavior change program (6 weeks)	1. STC 2. Delayed treatment	n=352 (cohorts of n=20-25)	PHQ-9^c^	6	13.9%	Unclear

Beatty et al [47]											
	Selective	PTSD^d^ Anxiety Depression	Adult cancer patients	Cancer Coping Online (CCO)	Self-guided web-based CBT^e^ (6 weeks)	1. CCO 2. Information only	n_1_=30 n_2_=30	DASS^f^ PSS^g^	4.5 7.5	8.3%	Yes

Christensen et al [48]											
	Indicated	GAD^h^ Depression	Young adults with mild GAD symptoms	iChill	Active website (CBT) + email (10 weeks)	1. Active website 2. Active +phone 3. Active +email 4. Control website 5. Control +phone	n_1_ = 111 n_2_ = 110 n_3_ = 113 n_4_ = 113 n_5_ = 111	GAD-7^i^ MINI^j^ CES-D^k^	6 12	52.69%	Yes

Christensen et al [55]											
	Indicated and selective	Depression	Adult Internet users with insomnia and subthreshold depression	SHUTi	Modular insomnia website (6 weeks)	1. SHUTi 2. Placebo website (HealthWatch)	n_1_=574 n_2_=575	PHQ-9 MINI	1.5 6	56.1%	Yes

Imamura et al [49]											
	Indicated	Depression	Workers with subthreshold depression	Internet CBT program (iCBT)	Guided stress management training (manga) (6 weeks)	1. iCBT 2. Information only	n_1_=381 n_2_=381	BDI-II^l^ WHO-CIDI^m^	12	32.9%	Yes







Jacobi et al [39]											
	Selective	Eating disorders	Female university students	StudentBodies (SB)	Structured CBT + discussion group (8 weeks)	1. SB 2. Waiting list	n_1_=50 n_2_=50	EDE-Q^n^ SCID^o^ EDI-2^p^	3	6.00%	Unclear

Jacobi et al [40]											
	Indicated	Eating disorders	Women with subthreshold eating disorders	StudentBodies+ (SB+)	Structured CBT + symptom checklist/ body image exercise (8 weeks)	1. SB+ 2. Waiting list	n_1_=64 n_2_=62	EDE-Q SCID	6	18.3%	Unclear

Mitchell et al [50]											
	Universal	Anxiety Depression	Adult Australian residents	Strength-Intervention	Self-guided text- and graphic-based interactive program (3 weeks)	1. Strength-Intervention 2. Placebo	n_1_=48 n_2_=54	DASS	3	78.4%	Yes

Mouthaan et al [51]											
	Indicated	PTSD Anxiety Depression	Injury patients	Trauma TIPS	Self-guided Internet-based CBT (30 min)	1. Trauma TIPS 2. Care as usual	n_1_=151 n_2_=149	HADS^q^ CAPS^r^ MINI	1 3 6 12	53.7%	Yes


Musiat et al [41]											
	Universal	Anxiety Depression Eating disorders	University students	Personality and Living of University Students (PLUS)	Automated transdiagnostic trait focused Web-based intervention (5x 20-40 min)	1. PLUS 2. Placebo	n_1_=519 n_2_=528	PHQ GAD-7 EDDS^s^	3	61.7%	Unclear






Powell et al [52]											
	Universal	GAD	Users of the UK National Health Service	MoodGYM	Self-directed CBT training (5 weeks)	1.MoodGYM 2. Waiting list	n_1_=1534 n_2_=1536	CES-D GAD-7	3	50.2%	Yes

Proudfoot et al [53]											
	Indicated	Anxiety Depression	Adults with mild to moderate anxiety or depression	myCompass	Automated intervention + symptom self-monitoring (7 weeks)	1. MyCompass 2. Attention control 3. Waiting list	n_1_=242 n_2_=248 n_3_=230	DASS	3	51.4%	Yes


Stice et al [42]											
	Selective	Eating disorders Depression	Female college student with body dissatisfaction	eBody Project (eBP)	Self-guided cognitive- behavioral program to change thin ideal (3 weeks)	1. eBP 2. BP 3. Video control 4. Brochure control	n_1_=19 n_2_=39 n_3_=29 n_4_=20	BDI^t^ EDDI^u^	12 24	4.7%	Unclear


Taylor et al [43]											
	Selective	Eating disorders Depression	College women with high weight/ shape concerns	StudentBodies (SB)	Structured CBT+ discussion group (8 weeks)	1. SB 2. Waiting list	n_1_=244 n_2_=236	CES-D EDE-I^v^ EDE-Q	12 24 36	12.3%	Unclear








Thompson et al [54]											
	Indicated	Depression	Mild- to moderately depressed epilepsy patients	Using Practice and Learning to Increase favorable thoughts (UPLIFT)	Telephone- and Web-based mindfulness and cognitive therapy (8 weeks)	1. UPLIFT 2. Waiting list	n_1_=64 n_2_=64	BDI mBDI^w^ NDDI-E^x^ PHQ	2 4	15.6%	Yes


Winzelberg et al [44]											
	Selective	Eating disorders	Female university students	Student Bodies (SB)	Structured CBT+ discussion group	1. SB 2. Waiting list	n_1_=31 n_2_=29	EDE-Q	3	26.7%	Yes

Zabinski et al [45]											
	Selective	Eating disorders	College age women	Chat room	Private chat room for moderated discussion (8 weeks)	1. Chat room 2. Waiting list	n_1_=30 n_2_=30	EDE-Q	4.5	3.3%	Yes

^a^Dropout-rate from baseline to the longest available follow-up.

^b^ITT: Intention-to-treat-analysis

^c^PHQ: Personal Health Questionnaire depression scale

^d^PTSD: Posttraumatic Stress Disorder

^e^CBT: Cognitive Behavioral Therapy

^f^DASS: Depression Anxiety Stress Scale

^g^PSS: PTSD Symptom Scale

^h^GAD: Generalized Anxiety Disorder

^i^GAD-7: Generalized Anxiety Disorder questionnaire – 7

^j^MINI: Mini-International Neuropsychiatric Interview

^k^CES-D: Center for Epidemiological Studies Depression scale

^l^BDI-II: Beck Depression Inventory II

^m^CIDI: WHO Composite International Diagnostic Interview (Web-based, self-administered version)

^n^EDE-Q: Eating Disorder Examination Questionnaire

^o^SCID: Structured Clinical Interview for DSM Disorders

^p^EDI-2: Eating Disorder Inventory

^q^HADS: Hospital Anxiety and Depression Scale

^r^CAPS: Clinician-Administered PTSD Scale

^s^EDDS: Eating Disorders Diagnostic Scale

^t^BDI: Beck Depression Inventory

^u^EDDI: Eating Disorder Diagnostic Interview

^v^EDE-I: Eating Disorder Examination Interview

^w^mBDI: Modified Beck Depression Inventory

^x^NDDI-E: Neurological Disorders Depression Inventory in Epilepsy

### Eating Disorders

The systematic search yielded 6 studies on eating disorders. Four evaluated the effectiveness of StudentBodies, an Internet-based intervention for young women at risk of developing an eating disorder or with subthreshold eating disorders. StudentBodies was originally developed and evaluated in the United States [43,44] and was later translated to the German language [39,40]. The program makes use of common CBT principles and includes a Web-based discussion group.

Also inspired by StudentBodies, Zabinski et al [45] developed a moderated synchronous group intervention for college-age women, called Chat Room. The main difference to StudentBodies is the integration of a synchronous communication chat room that enables participants to communicate more directly with each other.

Stice et al [42] developed and evaluated the eBody Project, an Internet-based version of a CBT-based eating disorder prevention group-program. eBody is a dissonance-based intervention encouraging young women to question the popular “thin” ideal.

### Depression

The search yielded 3 prevention programs focused on the prevention of depression. Surviving and Thriving with Cancer [46] is an assisted Web-based education course aimed to foster positive health behaviors in cancer survivors. In addition to depression, intervention effects on health conditions as nutrition, exercise, and sleep were examined.

The guided Internet-based CBT program (iCBT) by Imamura et al [49] conveys stress management skills to employees of 2 information technology (IT) companies with subthreshold depression. The intervention aims to prevent major depression episodes. It includes common CBT elements such as self-monitoring or relaxation techniques.

Thompson et al [54] adapted a mindfulness-based prevention program (UPLIFT) for epilepsy patients with mild-to-moderate depressive symptoms [56]. The telephone- and Web-based intervention makes use of psychoeducative principles (eg, knowledge about depression, importance of reinforcement) and mindfulness-based tools (eg, monitoring of thoughts).

The 6-week Web-based insomnia program SHUTi by Christensen et al [55] aims at the high co-occurrence of insomnia and depression. Overall, 1149 participants were recruited via the social network platform Facebook and randomized to either a CBT-based insomnia intervention or a control website (HealthWatch).

### Anxiety and Depression

Two studies focused on combined anxiety and depression. A self-guided intervention promoting well-being in a general population was tested by Mitchell et al [50]. In 3 weekly sessions, users completed either an interactive program focusing on strengths (intervention 1, based on positive psychology principles) or problem-solving skills (intervention 2). Users received feedback and email reminders.

MyCompass [53], a self-guided computer-delivered intervention, aims to foster self-management and self-monitoring skills in people with mild-to-moderate depression, anxiety, and stress symptoms. Content in the 12 modules was derived from CBT, interpersonal psychotherapy, problem-solving therapy, and positive psychology. Users are also provided with text messages or emails containing reminders and material on psychoeducation.

### Post-Traumatic Stress Disorder

There were 2 studies that focused on post-traumatic stress. The self-guided Trauma TIPS [51] aims to prevent PTSD in injury patients. CBT techniques such as psychoeducation, stress management, and in vivo exposure are presented in the 30-minute program alongside contact information for professional help and a Web forum for peer support.

Cancer Coping Online, a self-guided Web-based CBT program for reducing distress in patients currently receiving cancer treatment, was evaluated by Beatty et al [47]. The 6-session program mainly focuses on coping strategies taught via text, audios, and worksheets. Besides posttraumatic stress (cancer-specific distress), levels of depression and anxiety (general distress) were evaluated.

### Generalized Anxiety Disorder

The search yielded 2 studies on GAD. MoodGYM [52] is a fully automated Internet-based program that teaches CBT skills (eg, psychoeducation, relaxation, or mediation techniques) to improve mental well-being in a general population. Besides well-being, depression and GAD data were gathered. Because the mean depression score of the participants exceeded a clinical cutoff (and thus did not fulfill inclusion criteria), only GAD data were included in this review. Christensen et al [48] evaluated the program iChill, which aims to prevent GAD in young adults with mild GAD symptoms. iChill is an active website. The intervention makes use of multiple CBT tools as psychoeducation, relaxation, or toolkits. Telephone reminders for participants without therapeutic purpose were included in one trial arm.

### Common Mental Health Disorders

Musiat et al [41] developed a transdiagnostic trait-focused program (PLUS) aiming to prevent common mental disorders. Investigated disorders were depression, GAD, EDs, and alcohol misuse. The CBT-based program focuses on the identification of strengths and the consolidation of coping strategies and includes computerized feedback.

### Effectiveness

#### Primary Outcome Onset

Five studies reported incidence data allowing for the calculation of onset, IRR, and NNT (see Table 4). Christensen et al [48,55] did not observe significant group differences in onset. Imamura et al [49] reported a significant onset difference for the 12-month follow-up period (1.26% for the intervention group, 5.51% for the control group). Calculations yielded an IRR of 0.23 and a NNT of 23.48. Taylor et al [43] found an onset of 4.00% in the intervention group, compared with 6.60% in the control group. This yields an IRR of 0.63 and a NNT of 41.31. Thompson et al [54] found a 0% onset for the intervention group and a 10.70% onset for the control group. In accordance to the Cochrane Handbook for Systematic Reviews [57], a correction of 0.5 was added to the zero incidence in the intervention group, resulting an IRR of 0.09. The NNT was 9.33.

**Table 4:** Incidence, onset, incidence rate ratio (IRR), number needed to be treated (NNT)

Study	Disorder	Number of onsets		Follow-up	IRR^c^	NNT^d^
		IG^a^	CG^b^			
Christensen et al [55]	Depression	N=9 N_Total_= 224	N=13 N_Total_= 280	6 months	0.87	160
Imamura et al [49]	Depression	n=3 N_Total_= 239	n=15 N_Total_= 272	12 months	0.23	23.5
Thompson et al [54]	Depression	n=0^e^ N_Total_= 52	n=6 N_Total_= 56	2 months (pre–post)	0.09	9.3
Taylor et al [43]	Eating Disorders	n=8 N_Total_= 193	n=13 N_Total_= 198	12 months	0.63	41.3
Christensen et al [48]	GAD^f^	n=10 N_Total_= 171	n=6 N_Total_= 132	6 months	1.29	−76.8

^a^IG: intervention group

^b^CG: control group

^c^IRR: incidence rate ratios

^d^NNT: number needed to treat

^e^A correction of 0.5 was added to the zero incidence in the intervention group for IRR calculation.

^f^GAD: Generalized Anxiety Disorder

#### Secondary Outcome: Severity

Severity data were extracted for all included studies. Eleven studies found significant effects on symptom severity with small-to-large effect sizes (*d*=0.11 to *d*=0.76), indicating differential intervention effects on group, time, or interactions of both respectively [39-41,43,45,47,51-55].

**Figure 2:**
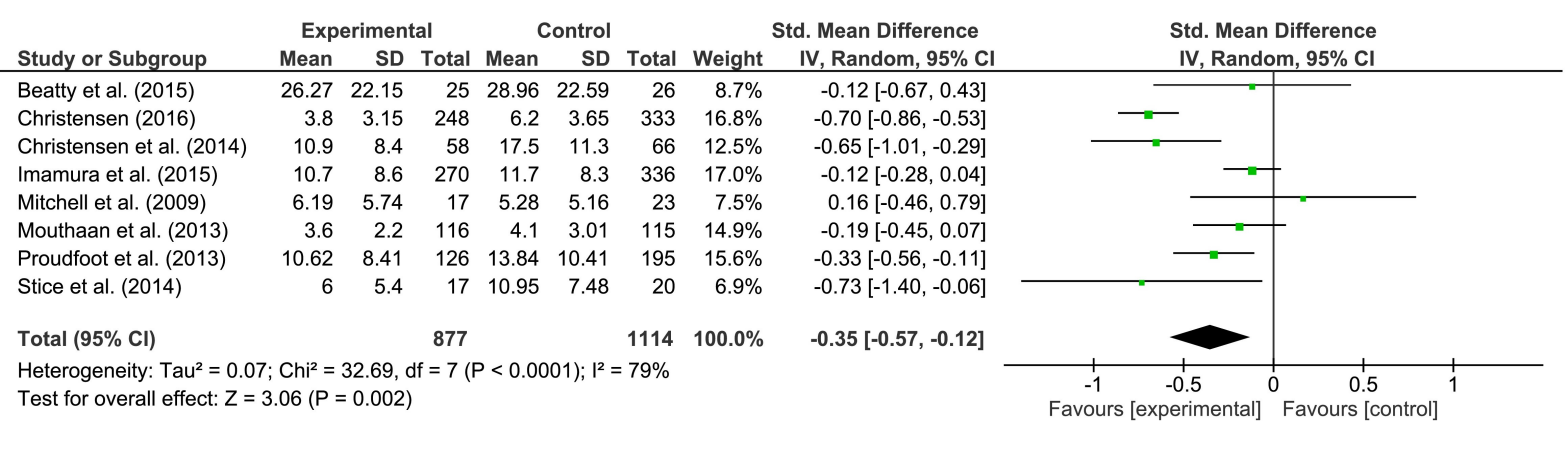
The effects of preventive interventions on symptom severity of depression at short-term FU—comparison experimental versus control group.

**Figure 3:**
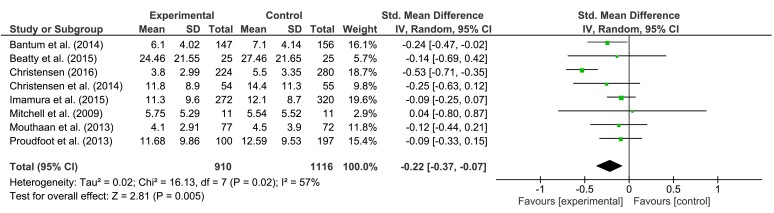
The effects of preventive interventions on symptom severity of depression at medium-term FU—comparison experimental versus control group.

**Figure 4:**
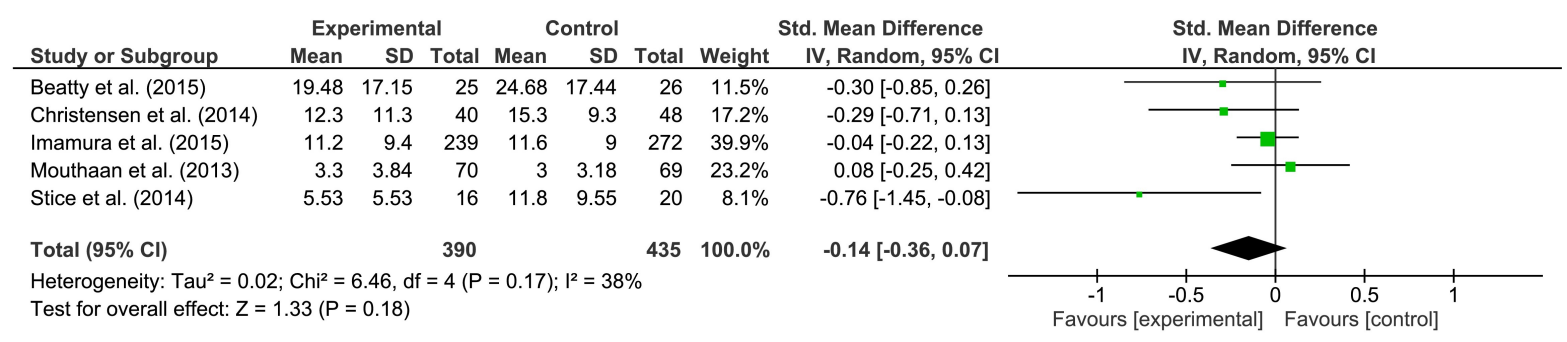
The effects of preventive interventions on symptom severity of depression at long-term FU—comparison experimental versus control group.

For the meta-analysis of depression interventions, we included studies with depression as a primary and secondary outcome. In cases of multiple active groups, only the main intervention sample was analyzed [48,50]. Results for short-, medium-, and long-term follow-up are presented in Figure 2,Figure 3, and Figure 4, respectively. In summary, pooled effect sizes, indicating a greater decrease in symptom severity for the intervention group, were small but significant.

For short-term follow-up, our calculations yielded an effect size of SMD = −0.35 (95% CI, −0.57 to −0.12, *P*=.002). Test of heterogeneity was significant (*P<*.001; *I*^2^= 79%). Effect size for medium-term follow-up was SMD = −0.22 (95% CI, −0.37 to −0.07), *P*=.005. Heterogeneity was significant (*P=*.02; *I*^2^= 57%). For long-term follow-up, an effect *SMD* = −0.14 (95% CI −0.36 to 0.07, *P*=.18) was found. Heterogeneity was not significant (*P=*.17; *I*^2^= 38%). According to Higgins et al [58], overall level of heterogeneity was moderate to high.

### Ongoing Mental eHealth Prevention Trials

An ICTRP search for ongoing trials yielded 570 records for 560 trials (years 2005-2015). Sixty-two records were selected as likely being relevant. Most of those were planned studies on symptom severity as a secondary outcome and with different study purposes. Eleven records aimed to assess severity data and had an explicit preventive goal. Targeted conditions were mostly mood and anxiety disorders. Seven studies planned to use clinical interviews for diagnostics or to explicitly gather incidence data. Three studies of those studies were already published, one of them is included in this review [49] and 2 were excluded because of high symptom severity or diagnosis (inclusion criteria 2) at baseline [59,60]. Another planned study on depression had been withdrawn before enrolment because of missing funding (ClinicalTrials.gov, registration no. NCT01080105). Of the remaining 3 records, one study is planned on the prevention of psychosis for people with psychotic like experiences (Australian New Zealand Clinical Trials Registry, registration no. ACTRN12612000963820). The other one concerns the prevention of depression in people with complicated grief (UMIN Clinical Trials Registry, registration no. UMIN000007331). Our own research group also conducts a clinical trial on the prevention of depression in the risk-group of back pain patients with subthreshold depressive symptoms, which is registered at the German Clinical Trials Register (registration no. DRKS00007960). Inspection study protocols emerging from the data base search yielded ongoing trials for the prevention of depression [61-64] and PTBS [65].

## Discussion

### Principal Findings

This review and meta-analysis systematically summarizes previous research on Internet-based interventions for the prevention of mental disorders. It therefore exceeds the informative value of existing reviews (eg, [23,67]) in terms of the range of included disorders and gives an extensive overview of the actual state of research.

Seventeen RCTs were included in this review and described in detail. Results are in line with previous meta-analyses, showing that indicated and selective prevention is more common than universal prevention [13] and that CBT is a frequently (sometimes even exclusively) used Internet-based intervention type (eg, [24,66]).

Quality assessment suggests that 5 included studies have a high risk of bias. Some biases are inevitable (eg, blinding not possible for psychological interventions). Others, such as biases due to inappropriate randomization, can and should be avoided. Of note, 3 studies were classified as having high risk of bias solely due to a serious flaw. Reasons for study classification as having a serious flaw included baseline differences between intervention and control groups [43,53], very low compliance with the intervention [50,53], and a very high dropout rate [50]. Concerning study dropout, the Cochrane guideline [36] provides a rather conservative appraisal when applied to IMIs [67]. When evaluating treatment dropout, a more differentiated perspective can be beneficial. In a recent meta-analysis, van Ballegooijen et al found that IMIs regularly have lower completer rates of total interventions when compared with face-to-face treatments (65.1% vs. 84.7%), but are equal in the percentage of average completed sessions (face-to-face CBT: 83.9% vs. iCBT: 80.8%) [68]. Above that, participants do not necessarily have to complete all sessions to benefit from IMIs. They may stop the treatment because they already obtained benefit, and therefore, these cases would represent a success, rather than a treatment dropout [69,70].

Three of 5 studies reporting incidence data provided evidence for a preventive effect of the investigated interventions [43,49,54]. One study failed to report the results of a diagnostic interview, which would have allowed calculating incidence rates [39]. The remaining studies by Christensen et al [48,55] did not find effects on reduction of new cases. However, secondary outcome measures of anxiety and depression symptoms showed positive effects. The included incidence studies differed in the length of follow up-periods. Thompson et al [54] only had a pre-post comparison. For the remaining studies, incidence reduction could be observed over a 12-month follow-up period [43,49], suggesting that preventive interventions have the potential for incidence reduction in the long term.

Nevertheless, severity data show positive effects of interventions in 11 of 17 studies with small-to-medium effect sizes. The best evidence was found for ED and depression. Beintner et al [23] previously conducted a meta-analytic review on most included ED studies, demonstrating mild-to-moderate effects on ED-related attitudes (*d*=0.15 to *d*=0.57). The only study not included in Beintners’ review [42] found similar effects. The Internet-based intervention was superior in the reduction of ED symptoms compared with the 2 control conditions (*d*=0.33 and *d*=0.19) at the long-term follow-up (1 year).

Our meta-analysis on IMIs for depression showed an overall small but significant reduction in symptom severity. As mentioned before, this demonstrates an effect of IMIs on the treatment of subclinical depression; a subsequent reduction of incidence can only be assumed, as most included studies did not report incidence data. Because of moderate to high levels of heterogeneity, the actual effect size values should be interpreted with caution. Nevertheless, heterogeneity results from estimates showing the same direction of effect favoring interventions over control groups.

In summary, evidence was found for effectiveness of interventions for EDs, depression, and anxiety. Internet-based interventions can be considered effective in reduction of subthreshold symptomatology and may also be suitable for preventing the onset of mental disorders over the long term. Depression and anxiety are of particular clinical relevance against the background of prevalence rates: as mentioned, anxiety disorders, insomnia, and major depression are the most common mental disorders in the European Union [5]. Although many IMIs include modules on sleep or relaxation (eg, [47,50]), Christensen et al [57] reported the first prevention RCT explicitly targeting insomnia resulting in a reduction in depressive symptomatology.

### Limitations

A number of potential limitations and challenges regarding this study should be acknowledged. As usual for reviews and meta-analyses, publication bias [71] must be assumed. This review included several studies reporting not expected or nonsignificant results; nevertheless, publication bias cannot be precluded. Furthermore, the search might have been confounded by language bias because only English and German papers were included. Fortunately, in contrast to publication bias, this only minimally impacts conclusions [72].

Another limitation concerns the inclusion of studies that reported mean scores only and did not clearly state that participants did not exceed clinical cutoffs at baseline (second exclusion criterion). This became evident after contacting authors to obtain raw data and subsequently computing incidence and onset rates if they had not already been reported. Four of those studies [73-76] had to be excluded because inspection of the raw data revealed that a considerable number of participants exceeded clinical cutoffs at baseline, even though the other eligibility criteria had been fulfilled. Unfortunately, only a few authors responded to our requests for additional information. Thus, it cannot be ruled out that this might also be the case for some included studies, that is, those which reported mean scores and did not provide raw data.

One major challenge of this broad review was the handling of variability between studies. Although heterogeneity was expected, and even welcomed to map out the broad scope of existing e-mental health prevention interventions, it must be taken into account when interpreting the findings. There are several sources of heterogeneity. First, this review was not restricted to one mental disorder but included a number of clinical conditions. Second, methods to determine the clinical status of participants, such as structured clinical interviews or self-report questionnaires, differed between studies. Third, intervention contents were different. As mentioned previously, most interventions were based on CBT, but other intervention types were included as well. Fourth, study design caused heterogeneity, due to different types of control groups, varying follow-up assessment periods and different sample sizes. For the meta-analysis, pooled effect sizes were calculated for depression and grouped into 3 follow-up periods. Nevertheless, different sample sizes can lead to overweighting of the larger size studies.

### Future Research

To gain insight into requirements for future research, limitations of the presented studies should be considered. First, the 5 included incidence studies were planned with preventive goals and used standardized clinical interviews for valid diagnosis. The remaining studies were often planned for other purposes (eg, improving well-being), and mental disorder symptoms were gathered by means of self-report questionnaires. Therefore, additional incidence studies using valid diagnostic instruments are needed, especially in light of the ICTRP search results, which revealed that very few incidence studies are planned in the near future.

Second, the evidence base is limited to a handful of disorder groups specifically EDs, depression and anxiety. Research could expand to the missing subfields, for which Internet-based prevention could be applicable. It is noteworthy that one ongoing trial targets prevention of psychosis [77].

Third, this is the first exhaustive review on Internet-based prevention for mental disorders in adults. One could expand the scope to additional domains and populations, for instance to relapse prevention in mentally ill persons. As there are already several studies on this topic (eg, [78,79]), a systematic review would be beneficial to provide an overview of the current state of research. Another potential field of study could be Internet-based prevention of mental disorders in children and adolescents. There is already one review on this topic [66], which is limited to depression and anxiety, but could be expanded to include other mental disorders.

Fourth, most Internet-based interventions included in this review had no additional human support component (ie, unguided). Although this results in a reduction of initial costs, it is also accompanied by a reduction of effectiveness [80]. To date, there is no study investigating whether or not this reduced effectiveness translates into an increase in costs over the long term, due to (for example) increased health care utilization or work incapacity days.

### Conclusions

Internet-based interventions can be effective in the primary prevention of mental disorders. The body of research is still limited to a few mental disorders (EDs, depression, anxiety disorders). Therefore, further high-quality studies are required, using standardized clinical interviews and gathering incidence data in long-term follow-ups. Because of the advantages of Internet-based interventions such as cost-effectiveness, availability, and flexibility [16,17], this can be a fruitful area for research. Content could be adapted for use with other disorders and populations. Furthermore, interventions that have been found to be effective in preventing certain mental disorders can and should be implemented into practice. Health care costs and personal, social, and financial burdens of the affected and society can consequently be reduced.

## Abbreviations

CBT: cognitive behavioral therapy
CENTRAL: Cochrane Central Register of Controlled Trials
CG: control group
ED: eating disorder
GAD: generalized anxiety disorder
ICTRP: International Clinical Trials Registry Platform
IG: intervention group
IMI: Internet- and mobile-based intervention
IT: Information technology
IRR: incidence rate ratio
MD: mean difference
MeSH: Medical Subject Heading
NNT: number needed to be treated
PRISMA: Preferred Reporting Items for Systematic Reviews and Meta-Analyses
PTSD: post-traumatic stress disorder
RCT: randomized controlled trial
SCID: structured clinical interview for DSM disorders

## References

[1] Kessler RC, Angermeyer M, Anthony JC, Demyttenaere K, Gasquet I, Gluzman S, Gureje O, Haro JM, Kawakami N, Karam A, Levinson D, Medina M, Oakley B, Posada-Villa J, Stein DJ, Adley T, Aguilar-Gaxiola S, Alonso J, Lee S, Heeringa S, Pennell B, Berglund P, Gruber MJ, Petukhova M, Chatterji S, Ustün TB (2007). Lifetime prevalence and age-of-onset distributions of mental disorders in the World Health Organization's World Mental Health Survey Initiative. World Psychiatry.

[2] Vos T, Flaxman AD, Naghavi M, Lozano R, Michaud C, Ezzati M, Shibuya K, Salomon JA, Abdalla S, Aboyans V, Abraham J, Ackerman I, Aggarwal R, Ahn SY, Ali MK, Alvarado M, Anderson HR, Anderson LM, Andrews KG, Atkinson C, Baddour LM, Bahalim AN, Barker-Collo S, Barrero LH, Bartels DH, Basáñez M, Baxter A, Bell ML, Benjamin EJ, Bennett D, Bernabé E, Bhalla K, Bhandari B, Bikbov B, Bin AA, Birbeck G, Black JA, Blencowe H, Blore JD, Blyth F, Bolliger I, Bonaventure A, Boufous S, Bourne R, Boussinesq M, Braithwaite T, Brayne C, Bridgett L, Brooker S, Brooks P, Brugha TS, Bryan-Hancock C, Bucello C, Buchbinder R, Buckle G, Budke CM, Burch M, Burney P, Burstein R, Calabria B, Campbell B, Canter CE, Carabin H, Carapetis J, Carmona L, Cella C, Charlson F, Chen H, Cheng AT, Chou D, Chugh SS, Coffeng LE, Colan SD, Colquhoun S, Colson KE, Condon J, Connor MD, Cooper LT, Corriere M, Cortinovis M, de Vaccaro KC, Couser W, Cowie BC, Criqui MH, Cross M, Dabhadkar KC, Dahiya M, Dahodwala N, Damsere-Derry J, Danaei G, Davis A, De LD, Degenhardt L, Dellavalle R, Delossantos A, Denenberg J, Derrett S, Des Jarlais DC, Dharmaratne SD, Dherani M, Diaz-Torne C, Dolk H, Dorsey ER, Driscoll T, Duber H, Ebel B, Edmond K, Elbaz A, Ali SE, Erskine H, Erwin PJ, Espindola P, Ewoigbokhan SE, Farzadfar F, Feigin V, Felson DT, Ferrari A, Ferri CP, Fèvre EM, Finucane MM, Flaxman S, Flood L, Foreman K, Forouzanfar MH, Fowkes FG, Franklin R, Fransen M, Freeman MK, Gabbe BJ, Gabriel SE, Gakidou E, Ganatra HA, Garcia B, Gaspari F, Gillum RF, Gmel G, Gosselin R, Grainger R, Groeger J, Guillemin F, Gunnell D, Gupta R, Haagsma J, Hagan H, Halasa YA, Hall W, Haring D, Haro JM, Harrison JE, Havmoeller R, Hay RJ, Higashi H, Hill C, Hoen B, Hoffman H, Hotez PJ, Hoy D, Huang JJ, Ibeanusi SE, Jacobsen KH, James SL, Jarvis D, Jasrasaria R, Jayaraman S, Johns N, Jonas JB, Karthikeyan G, Kassebaum N, Kawakami N, Keren A, Khoo J, King CH, Knowlton LM, Kobusingye O, Koranteng A, Krishnamurthi R, Lalloo R, Laslett LL, Lathlean T, Leasher JL, Lee YY, Leigh J, Lim SS, Limb E, Lin JK, Lipnick M, Lipshultz SE, Liu W, Loane M, Ohno SL, Lyons R, Ma J, Mabweijano J, MacIntyre MF, Malekzadeh R, Mallinger L, Manivannan S, Marcenes W, March L, Margolis DJ, Marks GB, Marks R, Matsumori A, Matzopoulos R, Mayosi BM, McAnulty JH, McDermott MM, McGill N, McGrath J, Medina-Mora ME, Meltzer M, Mensah GA, Merriman TR, Meyer A, Miglioli V, Miller M, Miller TR, Mitchell PB, Mocumbi AO, Moffitt TE, Mokdad AA, Monasta L, Montico M, Moradi-Lakeh M, Moran A, Morawska L, Mori R, Murdoch ME, Mwaniki MK, Naidoo K, Nair MN, Naldi L, Narayan KM, Nelson PK, Nelson RG, Nevitt MC, Newton CR, Nolte S, Norman P, Norman R, O'Donnell M, O'Hanlon S, Olives C, Omer SB, Ortblad K, Osborne R, Ozgediz D, Page A, Pahari B, Pandian JD, Rivero AP, Patten SB, Pearce N, Padilla RP, Perez-Ruiz F, Perico N, Pesudovs K, Phillips D, Phillips MR, Pierce K, Pion S, Polanczyk GV, Polinder S, Pope CA, Popova S, Porrini E, Pourmalek F, Prince M, Pullan RL, Ramaiah KD, Ranganathan D, Razavi H, Regan M, Rehm JT, Rein DB, Remuzzi G, Richardson K, Rivara FP, Roberts T, Robinson C, De Leòn FR, Ronfani L, Room R, Rosenfeld LC, Rushton L, Sacco RL, Saha S, Sampson U, Sanchez-Riera L, Sanman E, Schwebel DC, Scott JG, Segui-Gomez M, Shahraz S, Shepard DS, Shin H, Shivakoti R, Singh D, Singh GM, Singh JA, Singleton J, Sleet DA, Sliwa K, Smith E, Smith JL, Stapelberg NJ, Steer A, Steiner T, Stolk WA, Stovner LJ, Sudfeld C, Syed S, Tamburlini G, Tavakkoli M, Taylor HR, Taylor JA, Taylor WJ, Thomas B, Thomson WM, Thurston GD, Tleyjeh IM, Tonelli M, Towbin JA, Truelsen T, Tsilimbaris MK, Ubeda C, Undurraga EA, van der Werf MJ, van Os J, Vavilala MS, Venketasubramanian N, Wang M, Wang W, Watt K, Weatherall DJ, Weinstock MA, Weintraub R, Weisskopf MG, Weissman MM, White RA, Whiteford H, Wiersma ST, Wilkinson JD, Williams HC, Williams SR, Witt E, Wolfe F, Woolf AD, Wulf S, Yeh P, Zaidi AK, Zheng ZJ, Zonies D, Lopez AD, Murray CJ, AlMazroa MA, Memish ZA (2012). Years lived with disability (YLDs) for 1160 sequelae of 289 diseases and injuries 1990-2010: a systematic analysis for the Global Burden of Disease Study 2010. Lancet.

[3] Walker ER, McGee RE, Druss BG (2015). Mortality in mental disorders and global disease burden implications: a systematic review and meta-analysis. JAMA Psychiatry.

[4] Gustavsson A, Svensson M, Jacobi F, Allgulander C, Alonso J, Beghi E, Dodel R, Ekman M, Faravelli C, Fratiglioni L, Gannon B, Jones DH, Jennum P, Jordanova A, Jönsson L, Karampampa K, Knapp M, Kobelt G, Kurth T, Lieb R, Linde M, Ljungcrantz C, Maercker A, Melin B, Moscarelli M, Musayev A, Norwood F, Preisig M, Pugliatti M, Rehm J, Salvador-Carulla L, Schlehofer B, Simon R, Steinhausen HC, Stovner LJ, Vallat JM, Van den Bergh P, van Os J, Vos P, Xu W, Wittchen H, Jönsson B, Olesen J, CDBE2010Study Group (2011). Cost of disorders of the brain in Europe 2010. Eur Neuropsychopharmacol.

[5] Wittchen HU, Jacobi F, Rehm J, Gustavsson A, Svensson M, Jönsson B, Olesen J, Allgulander C, Alonso J, Faravelli C, Fratiglioni L, Jennum P, Lieb R, Maercker A, van Os J, Preisig M, Salvador-Carulla L, Simon R, Steinhausen HC (2011). The size and burden of mental disorders and other disorders of the brain in Europe 2010. Eur Neuropsychopharmacol.

[6] Gordon RS (1983). An operational classification of disease prevention. Public Health Rep.

[7] First MB, Spitzer RL, Gibbon M, Williams JB (2002). The Structured Clinical Interview for DSM-IV Axis I Disorders (SCID-I) and the Structured Clinical Interview for DSM-IV Axis II Disorders (SCID-II).

[8] Sheehan DV, Lecrubier Y, Sheehan KH, Amorim P, Janavs J, Weiller E, Hergueta T, Baker R, Dunbar GC (1998). The Mini-International Neuropsychiatric Interview (M.I.N.I.): the development and validation of a structured diagnostic psychiatric interview for DSM-IV and ICD-10. J Clin Psychiatry.

[9] Cuijpers P (2009). Prevention: an achievable goal in personalized medicine. Dialogues Clin Neurosci.

[10] Cuijpers P, Van Straten A, Smit F (2005). Preventing the incidence of new cases of mental disorders: a meta-analytic review. J Nerv Ment Dis.

[11] Cuijpers P, van Straten A, Smit F, Mihalopoulos C, Beekman A (2008). Preventing the onset of depressive disorders: a meta-analytic review of psychological interventions. Am J Psychiatry.

[12] Stice E, Shaw H, Marti CN (2007). A meta-analytic review of eating disorder prevention programs: encouraging findings. Annu Rev Clin Psychol.

[13] van Zoonen K, Buntrock C, Ebert DD, Smit F, Reynolds CF 3rd, Beekman AT, Cuijpers P (2014). Preventing the onset of major depressive disorder: a meta-analytic review of psychological interventions. Int J Epidemiol.

[14] Muñoz RF, Cuijpers P, Smit F, Barrera AZ, Leykin Y (2010). Prevention of major depression. Annu Rev Clin Psychol.

[15] Hayes JF, Maughan DL, Grant-Peterkin H (2016). Interconnected or disconnected? Promotion of mental health and prevention of mental disorder in the digital age. Br J Psychiatry.

[16] Cuijpers P, van Straten A, Warmerdam L, van Rooy MJ (2010). Recruiting participants for interventions to prevent the onset of depressive disorders: possible ways to increase participation rates. BMC Health Serv Res.

[17] Donker T, Blankers M, Hedman E, Ljótsson B, Petrie K, Christensen H (2015). Economic evaluations of Internet interventions for mental health: a systematic review. Psychol Med.

[18] Warmerdam L, Smit F, van Straten A, Riper H, Cuijpers P (2010). Cost-utility and cost-effectiveness of internet-based treatment for adults with depressive symptoms: randomized trial. J Med Internet Res.

[19] Titov N, Dear BF, Ali S, Zou JB, Lorian CN, Johnston L, Terides MD, Kayrouz R, Klein B, Gandy M, Fogliati VJ (2015). Clinical and cost-effectiveness of therapist-guided internet-delivered cognitive behavior therapy for older adults with symptoms of depression: a randomized controlled trial. Behav Ther.

[20] Ybarra ML, Eaton WW (2005). Internet-based mental health interventions. Ment Health Serv Res.

[21] Internet Society.

[22] Barak A, Klein B, Proudfoot JG (2009). Defining internet-supported therapeutic interventions. Ann Behav Med.

[23] Beintner I, Jacobi C, Taylor CB (2012). Effects of an Internet-based prevention programme for eating disorders in the USA and Germany--a meta-analytic review. Eur Eat Disord Rev.

[24] Schlegl S, Bürger C, Schmidt L, Herbst N, Voderholzer U (2015). The potential of technology-based psychological interventions for anorexia and bulimia nervosa: a systematic review and recommendations for future research. J Med Internet Res.

[25] Rooke S, Thorsteinsson E, Karpin A, Copeland J, Allsop D (2010). Computer-delivered interventions for alcohol and tobacco use: a meta-analysis. Addiction.

[26] Champion KE, Newton NC, Barrett EL, Teesson M (2013). A systematic review of school-based alcohol and other drug prevention programs facilitated by computers or the internet. Drug Alcohol Rev.

[27] Tait RJ, Spijkerman R, Riper H (2013). Internet and computer based interventions for cannabis use: a meta-analysis. Drug Alcohol Depend.

[28] Hustad JT, Barnett NP, Borsari B, Jackson KM (2010). Web-based alcohol prevention for incoming college students: a randomized controlled trial. Addict Behav.

[29] Paschall MJ, Antin T, Ringwalt CL, Saltz RF (2011). Evaluation of an Internet-based alcohol misuse prevention course for college freshmen: findings of a randomized multi-campus trial. Am J Prev Med.

[30] Liberati A, Altman DG, Tetzlaff J, Mulrow C, Gøtzsche PC, Ioannidis JP, Clarke M, Devereaux PJ, Kleijnen J, Moher D (2009). The PRISMA Statement for Reporting Systematic Reviews and Meta-Analyses of Studies That Evaluate Health Care Interventions: Explanation and Elaboration. Ann Intern Med.

[31] Sander L, Rausch L, Baumeister H (2016). Effectiveness of Internet- and mobile-based psychological interventions for the prevention of mental disorders: a systematic review and meta-analysis protocol. Syst Rev.

[32] First MB, Gibbon M, Hilsenroth MJ, Segal DL, Hersen M (2004). The Structured Clinical Interview for DSM-IV Axis I Disorders (SCID-I)the Structured Clinical Interview for DSM-IV Axis II Disorders (SCID-II). Comprehensive Handbook of Psychological Assessment, Volume 2, Personality Assessment.

[33] Beck AT, Steer RA, Brown GK (1996). Manual for the Beck Depression Inventory-II.

[34] Hamilton M (1960). A rating scale for depression. J Neurol Neurosurg Psychiatry.

[35] Kampling H, Baumeister H, Jäckel WH, Mittag O (2014). Prevention of depression in chronically physically ill adults. Cochrane Database Syst Rev.

[36] Furlan AD, Pennick V, Bombardier C, van Tudler M, Editorial Board‚ Cochrane Back Review Group (2009). 2009 updated method guidelines for systematic reviews in the Cochrane Back Review Group. Spine (Phila Pa 1976).

[37] Higgins JP, Thompson SG (2002). Quantifying heterogeneity in a meta-analysis. Stat Med.

[38] Sterne JA, Sutton AJ, Ioannidis JP, Terrin N, Jones DR, Lau J, Carpenter J, Rücker G, Harbord RM, Schmid CH, Tetzlaff J, Deeks JJ, Peters J, Macaskill P, Schwarzer G, Duval S, Altman DG, Moher D, Higgins JP (2011). Recommendations for examining and interpreting funnel plot asymmetry in meta-analyses of randomised controlled trials. BMJ.

[39] Jacobi C, Morris L, Beckers C, Bronisch-Holtze J, Winter J, Winzelberg AJ, Taylor CB (2007). Maintenance of internet-based prevention: a randomized controlled trial. Int J Eat Disord.

[40] Jacobi C, Völker U, Trockel MT, Taylor CB (2012). Effects of an Internet-based intervention for subthreshold eating disorders: a randomized controlled trial. Behav Res Ther.

[41] Musiat P, Conrod P, Treasure J, Tylee A, Williams C, Schmidt U (2014). Targeted prevention of common mental health disorders in university students: randomised controlled trial of a transdiagnostic trait-focused web-based intervention. PLoS One.

[42] Stice E, Durant S, Rohde P, Shaw H (2014). Effects of a prototype Internet dissonance-based eating disorder prevention program at 1- and 2-year follow-up. Health Psychol.

[43] Taylor CB, Bryson S, Luce KH, Cunning D, Doyle AC, Abascal LB, Rockwell R, Dev P, Winzelberg AJ, Wilfley DE (2006). Prevention of eating disorders in at-risk college-age women. Arch Gen Psychiatry.

[44] Winzelberg AJ, Eppstein D, Eldredge KL, Wilfley D, Dasmahapatra R, Dev P, Taylor CB (2000). Effectiveness of an Internet-based program for reducing risk factors for eating disorders. J Consult Clin Psychol.

[45] Zabinski MF, Wilfley DE, Calfas KJ, Winzelberg AJ, Taylor CB (2004). An interactive psychoeducational intervention for women at risk of developing an eating disorder. J Consult Clin Psychol.

[46] Bantum EO, Albright CL, White KK, Berenberg JL, Layi G, Ritter PL, Laurent D, Plant K, Lorig K (2014). Surviving and thriving with cancer using a Web-based health behavior change intervention: randomized controlled trial. J Med Internet Res.

[47] Beatty L, Koczwara B, Wade T (2016). Evaluating the efficacy of a self-guided Web-based CBT intervention for reducing cancer-distress: a randomised controlled trial. Support Care Cancer.

[48] Christensen H, Batterham P, Mackinnon A, Griffiths KM, Kalia HK, Kenardy J, Gosling J, Bennett K (2014). Prevention of generalized anxiety disorder using a web intervention, iChill: randomized controlled trial. J Med Internet Res.

[49] Imamura K, Kawakami N, Furukawa TA, Matsuyama Y, Shimazu A, Umanodan R, Kawakami S, Kasai K (2015). Does Internet-based cognitive behavioral therapy (iCBT) prevent major depressive episode for workers? A 12-month follow-up of a randomized controlled trial. Psychol Med.

[50] Mitchell J, Stanimirovic R, Klein B, Vella-Brodrick D (2009). A randomised controlled trial of a self-guided internet intervention promoting well-being. Comput Human Behav.

[51] Mouthaan J, Sijbrandij M, de Vries G, Reitsma JB, van de Schoot R, Goslings JC, Luitse JS, Bakker FC, Gersons BP, Olff M (2013). Internet-based early intervention to prevent posttraumatic stress disorder in injury patients: randomized controlled trial. J Med Internet Res.

[52] Powell J, Hamborg T, Stallard N, Burls A, McSorley J, Bennett K, Griffiths KM, Christensen H (2012). Effectiveness of a web-based cognitive-behavioral tool to improve mental well-being in the general population: randomized controlled trial. J Med Internet Res.

[53] Proudfoot J, Clarke J, Birch MR, Whitton AE, Parker G, Manicavasagar V, Harrison V, Christensen H, Hadzi-Pavlovic D (2013). Impact of a mobile phone and web program on symptom and functional outcomes for people with mild-to-moderate depression, anxiety and stress: a randomised controlled trial. BMC Psychiatry.

[54] Thompson NJ, Patel AH, Selwa LM, Stoll SC, Begley CE, Johnson EK, Fraser RT (2015). Expanding the efficacy of Project UPLIFT: Distance delivery of mindfulness-based depression prevention to people with epilepsy. J Consult Clin Psychol.

[55] Christensen H, Batterham PJ, Gosling JA, Ritterband LM, Griffiths KM, Thorndike FP, Glozier N, O'Dea B, Hickie IB, Mackinnon AJ (2016). Effectiveness of an online insomnia program (SHUTi) for prevention of depressive episodes (the GoodNight Study): a randomised controlled trial. The Lancet Psychiatry.

[56] Thompson NJ, Walker ER, Obolensky N, Winning A, Barmon C, Diiorio C, Compton MT (2010). Distance delivery of mindfulness-based cognitive therapy for depression: project UPLIFT. Epilepsy Behav.

[57] Higgins J, Green S (2011). The Cochrane Collaboration.

[58] Higgins JP, Thompson SG, Deeks JJ, Altman DG (2003). Measuring inconsistency in meta-analyses. BMJ.

[59] Kersting A, Dölemeyer R, Steinig J, Walter F, Kroker K, Baust K, Wagner B (2013). Brief Internet-based intervention reduces posttraumatic stress and prolonged grief in parents after the loss of a child during pregnancy: a randomized controlled trial. Psychother Psychosom.

[60] van Ballegooijen W, Riper H, Klein B, Ebert DD, Kramer J, Meulenbeek P, Cuijpers P (2013). An Internet-based guided self-help intervention for panic symptoms: randomized controlled trial. J Med Internet Res.

[61] Buntrock C, Ebert DD, Lehr D, Cuijpers P, Riper H, Smit F, Berking M (2014). Evaluating the efficacy and cost-effectiveness of web-based indicated prevention of major depression: design of a randomised controlled trial. BMC Psychiatry.

[62] Almeida OP, MacLeod C, Ford A, Grafton B, Hirani V, Glance D, Holmes E (2014). Cognitive bias modification to prevent depression (COPE): study protocol for a randomised controlled trial. Trials.

[63] Jones BA, Griffiths KM, Christensen H, Ellwood D, Bennett K, Bennett A (2013). Online cognitive behaviour training for the prevention of postnatal depression in at-risk mothers: a randomised controlled trial protocol. BMC Psychiatry.

[64] Klein JP, Berger T, Schröder J, Späth C, Meyer B, Caspar F, Lutz W, Greiner W, Hautzinger M, Rose M, Gräfe V, Hohagen F, Andersson G, Vettorazzi E, Moritz S (2013). The EVIDENT-trial: protocol and rationale of a multicenter randomized controlled trial testing the effectiveness of an online-based psychological intervention. BMC Psychiatry.

[65] Freedman SA, Dayan E, Kimelman YB, Weissman H, Eitan R (2015). Early intervention for preventing posttraumatic stress disorder: an Internet-based virtual reality treatment. Eur J Psychotraumatol.

[66] Calear A, Christensen H (2010). Review of internet-based prevention and treatment programs for anxiety and depression in children and adolescents. Med J Aust.

[67] Melville KM, Casey LM, Kavanagh DJ (2010). Dropout from Internet-based treatment for psychological disorders. Br J Clin Psychol.

[68] van Ballegooijen W, Cuijpers P, van Straten A, Karyotaki E, Andersson G, Smit JH, Riper H (2014). Adherence to Internet-based and face-to-face cognitive behavioural therapy for depression: a meta-analysis. PLoS One.

[69] Hilvert-Bruce Z, Rossouw PJ, Wong N, Sunderland M, Andrews G (2012). Adherence as a determinant of effectiveness of internet cognitive behavioural therapy for anxiety and depressive disorders. Behav Res Ther.

[70] Clarke G, Kelleher C, Hornbrook M, Debar L, Dickerson J, Gullion C (2009). Randomized effectiveness trial of an Internet, pure self-help, cognitive behavioral intervention for depressive symptoms in young adults. Cogn Behav Ther.

[71] Dickersin K (1990). The existence of publication bias and risk factors for its occurrence. JAMA.

[72] Wright RW, Brand RA, Dunn W, Spindler KP (2007). How to write a systematic review. Clin Orthop Relat Res.

[73] Bolier L, Haverman M, Kramer J, Westerhof GJ, Riper H, Walburg JA, Boon B, Bohlmeijer E (2013). An Internet-based intervention to promote mental fitness for mildly depressed adults: randomized controlled trial. J Med Internet Res.

[74] Eisma MC, Boelen PA, van den Bout J, Stroebe W, Schut HA, Lancee J, Stroebe MS (2015). Internet-Based Exposure and Behavioral Activation for Complicated Grief and Rumination: A Randomized Controlled Trial. Behav Ther.

[75] Drozd F, Skeie LG, Kraft P, Kvale D (2014). A web-based intervention trial for depressive symptoms and subjective well-being in patients with chronic HIV infection. AIDS Care.

[76] van der Houwen K, Schut H, van den Bout J, Stroebe M, Stroebe W (2010). The efficacy of a brief internet-based self-help intervention for the bereaved. Behav Res Ther.

[77] Stafford E, Hides L, Kavanagh DJ (2015). The acceptability, usability and short-term outcomes of Get Real: A web-based program for psychotic-like experiences (PLEs). Internet Interventions.

[78] Fichter MM, Quadflieg N, Nisslmüller K, Lindner S, Osen B, Huber T, Wünsch-Leiteritz W (2012). Does internet-based prevention reduce the risk of relapse for anorexia nervosa?. Behav Res Ther.

[79] Holländare F, Johnsson S, Randestad M, Tillfors M, Carlbring P, Andersson G, Engström I (2011). Randomized trial of Internet-based relapse prevention for partially remitted depression. Acta Psychiatr Scand.

[80] Baumeister H, Reichler L, Munzinger M, Lin J (2014). The impact of guidance on Internet-based mental health interventions — A systematic review. Internet Interv.

