# Metadata (ORCID) Correction: Effectiveness of Internet-Based Interventions for the Prevention of Mental Disorders: A Systematic Review and Meta-Analysis

**DOI:** 10.2196/mental.6532

**Published:** 2016-08-25

**Authors:** Lasse Sander, Leonie Rausch, Harald Baumeister

**Affiliations:** ^1^ Institute of Psychology Department of Rehabilitation Psychology and Psychotherapy University of Freiburg Freiburg Germany; ^2^ Medical Faculty Department of Medical Psychology and Medical Sociology University of Freiburg Freiburg Germany; ^3^ Institute of Psychology and Education Department of Clinical Psychology and Psychotherapy University of Ulm Ulm Germany

The authors of “Effectiveness of Internet-Based Interventions for the Prevention of Mental Disorders: A Systematic Review and Meta-Analysis” (JMIR Mental Health 3(3):e38) inadvertently provided an incorrect ORCID for author Lasse Sander.

The ORCID originally published for Lasse Sander belonged to another author with the same name. The correct ORCID is: 0000-0002-4222-9837. This error has been corrected in the online version of the paper on the JMIR website on August 25, 2016, together with publishing this correction notice. The correction was made before submission to PubMed Central.

